# Increased levels of plasma total tau in adult Down syndrome

**DOI:** 10.1371/journal.pone.0188802

**Published:** 2017-11-30

**Authors:** Takashi Kasai, Harutsugu Tatebe, Masaki Kondo, Ryotaro Ishii, Takuma Ohmichi, Wing Tung Esther Yeung, Masafumi Morimoto, Tomohiro Chiyonobu, Naoto Terada, David Allsop, Masanori Nakagawa, Toshiki Mizuno, Takahiko Tokuda

**Affiliations:** 1 Department of Neurology, Kyoto Prefectural University of Medicine, Kyoto, Japan; 2 Department of Zaitaku (Homecare) Medicine, Kyoto Prefectural University of Medicine, Kyoto, Japan; 3 Maastricht University Medical Centre, Maastricht, the Netherlands; 4 Department of Pediatrics, Kyoto Prefectural University of Medicine, Graduate School of Medical Science, Kyoto, Japan; 5 North Medical Center, Kyoto Prefectural University of Medicine, Kyoto, Japan; 6 Hananoki Medical Welfare Center, Kyoto, Japan; 7 Division of Biomedical and Life Sciences, Faculty of Health and Medicine, Lancaster University, Lancaster, United Kingdom; 8 Department of Molecular Pathobiology of Brain Diseases, Kyoto Prefectural University of Medicine, Kyoto, Japan; Nathan S Kline Institute, UNITED STATES

## Abstract

Down syndrome (DS) is the most prevalent chromosomal abnormality. Early-onset dementia with the pathology of Alzheimer’s disease (AD) frequently develops in DS. Reliable blood biomarkers are needed to support the diagnosis for dementia in DS, since positron emission tomography or cerebrospinal fluid sampling is burdensome, particularly for patients with DS. Plasma t-tau is one of the established biomarkers for the diagnosis of AD, suggesting the potential value of t-tau as a biomarker for dementia in DS. The aim of this study was to assess and compare plasma levels of t-tau in adults with DS and in an age-matched control population. In this study, plasma levels of t-tau in 21 patients with DS and 22 control participants were measured by an ultrasensitive immunoassay technology, the single-molecule immunoarray (Simoa) method. We observed significantly increased plasma t-tau levels in the DS group (mean ± standard deviation (SD) = 0.643±0.493) compared to those in the control group (mean ± SD = 0.470±0.232): P = 0.0050. Moreover, age dependent correlation of plasma t-tau was only found in the DS group, and not in the control group. These findings suggest that elevated plasma t-tau levels reflect AD pathology and therefore have potential as an objective biomarker to detect dementia in adult DS.

## Introduction

Down syndrome (DS) is the most frequently occurring chromosomal abnormality in humans and affects between 1 in 700 babies born [1]. Decades ago, most people with Down syndrome (DS) did not live to old age. However, their average life expectancy has now been improved dramatically and exceeds 50 years in developed countries due to better health care [2, 3]. This general aging of the DS population is an important issue for neurologists because there is a greatly increased risk of developing dementia in DS individuals over 40 years old [4]. Pathological brain changes of aged individuals with DS are almost identical to those of patients with Alzheimer’s disease (AD), consisting of both senile plaques and neurofibrillary tangles composed of amyloid β (Aβ) and phosphorylated tau, respectively [5]. The mechanisms behind this are thought to be overexpression of *APP* located on chromosome 21 that leads overproduction of Aβ [5] as well as overexpression of *Dyrk1A* and *RCAN1* also located on chromosome 21 which are both involved in hyperphosphorylation of tau [6–8]. In this context, adults with DS can be regarded as a group that is strongly vulnerable for the development of AD. Based on these considerations, people with DS have even been recognized as having presymptomatic AD, according to International Working Group-2 criteria, similar to carriers of autosomal dominant mutations in *PSEN1*, *PSEN2*, or *APP* [9].

The prevalence of dementia in DS was reported to be between 10% and 25% in the 40 to 49 years age group, between 20% and 50% in the 50 to 59 years age group, and between 30% and 75% in those older than 60 years [10–14]. This diversity of prevalence is caused by the difficulty of diagnosing dementia in people with DS, since their baseline cognitive abilities can vary considerably. Moreover, the diagnosis of dementia in DS cannot be completely relied upon even when dementia screening instruments for people with intellectual disabilities are used. The reason for this doubt is because such instruments are based on observer-rated questionnaires and so are not free from observer bias of caregivers.

Considering the similarity between dementia in DS and AD, approaches for objective detection of dementia in DS could be aided by use of biomarkers employed for the diagnosis of AD. In fact, some research groups have recently detected age-dependent accumulation of Aβ and tau in the brains of adults with DS by using positron emission tomography [15–17]. In addition, another group has proposed the utility of Aβ peptide and total-tau (t-tau) concentrations in cerebrospinal fluid (CSF), which are regarded as core biomarkers in AD, for the screening of dementia in DS [18]. However, these approaches cannot be utilized for general medical practice due to expensive, highly invasive or labor-intensive procedures. This could be overcome by the use of peripheral blood biomarkers. Plasma Aβ species have been expected to discriminate between demented and non-demented individuals with DS. In fact, many of studies measuring plasma Aβ species in DS agreed that plasma levels of Aβ species in DS were significantly higher than those in normal individuals, mainly because of overproduction of *APP* [19]. However, it is still controversial whether plasma Aβ species can serve as reliable biomarkers for diagnosis dementia in DS [20]. According to a large scale meta-analysis, plasma t-tau was the only blood biomarker with sufficient effect size in the AD field [21]. To our knowledge, plasma t-tau has been little investigated in DS [22]. The aim of this study was to assess and compare plasma levels of t-tau in adults with DS and in an age-matched control population.

Reproducible measurements of plasma t-tau have been difficult because of its very low concentration in peripheral blood. However, the recent development of an ultrasensitive immunoassay technology, the single-molecule immunoarray (Simoa) method (Quanterix, Inc), provides a sensitivity that is approximately 1000 times improved compared with conventional assays for plasma t-tau detection, making it feasible to use for reliable case-control studies [23–25]. Considering this recent development, we measured t-tau in plasma by using the Simoa technique.

## Materials and methods

### Study design, ethics statement, and subject recruitment

All study subjects provided written informed consent before participation and the study protocols were approved by the University Ethics Committee (RBMR-C-1226 for participants with DS and ERB-G-12 for controls, Kyoto Prefectural University of Medicine, Kyoto, Japan). Informed consent in the DS group was obtained from the patient when possible and from the nearest relative. Study procedures were designed and performed in accordance with the Declaration of Helsinki. We enrolled 21 adult patients with DS (DS group) from the registration for DS in Kyoto Prefectural University of Medicine and Hananoki Medical Welfare Center, from February 2013 to January 2017. Age matched healthy volunteers or patients presenting no neurological symptoms within three month prior to sample collection were enrolled as the control group. Individuals with any chromosomal abnormality, neurodegenerative diseases, or family history of AD in their first degree relatives were excluded from this group. Plasma from 22 individuals of the control group was obtained from another registration of Kyoto Prefectural University of Medicine during the aforementioned period. Plasma samples were taken through venous puncture and a total of 8 ml of blood was collected in EDTA-containing tubes. After collection, plasma was separated by centrifugation for 10 min at 3000 rpm and distributed in polypropylene vials. Fresh samples obtained from the enrolled subjects were immediately stored at -80°C until analysis.

Social maturity in the DS group was estimated as ‘social ages’ using the social maturity scale revised (S-M) (Nihonbuknasha, Tokyo), which is a social maturity scale developed for the Japanese based on the Vineland Social Maturity Scale [26]. Patients with DS were also systematically screened using Dementia Screening Questionnaires for Individuals with Intellectual Disabilities [27] (DSQIID, Japanese edition was obtained from the following website; http://www.nozomi.go.jp/publication/kiyou_04.htm). DSQIID scores behaviors and symptoms suggestive of dementia with 43 questions in its part 2 section, and compares current levels of function to those of baseline ability with 10 questions in its part 3 section. A total score, combined from part 2 and part 3, of >20 is considered the threshold [27]. Scores from part 1 of DSQIID, representing baseline “best” ability, were not used in the current study. These questionnaires were completed by family or caregivers.

### Measurement of t-tau

Plasma t-tau was measured with the Human Total Tau kit (research use only grade, Quanterix, Lexington, MA) on the Simoa HD-1 analyzer (Quanterix), in accordance with an updated version of the assay described previously that uses a monoclonal capture antibody that reacts with a linear epitope in the midregion of all tau isoforms and a detection antibody that reacts with a linear epitope in the N-terminus of t-tau [28] [24]. All samples were analyzed in duplicate on one occasion. (Intra-assay coefficients of variance of the measurements was 5.03%.) All cases and controls were evenly distributed on the examination.

### Statistics

The level of significance was set at P<0.05. A comparison between the two independent groups was performed using the Mann-Whitney U test. The Chi-square test was used to evaluate the statistical significance in categorical variables. The correlation analysis was performed using Spearman’s rank correlation coefficient test. All analyses were performed using SPSS for Windows version 23 software (IBM Japan Ltd, Tokyo, Japan).

## Results

**Fig 1:**
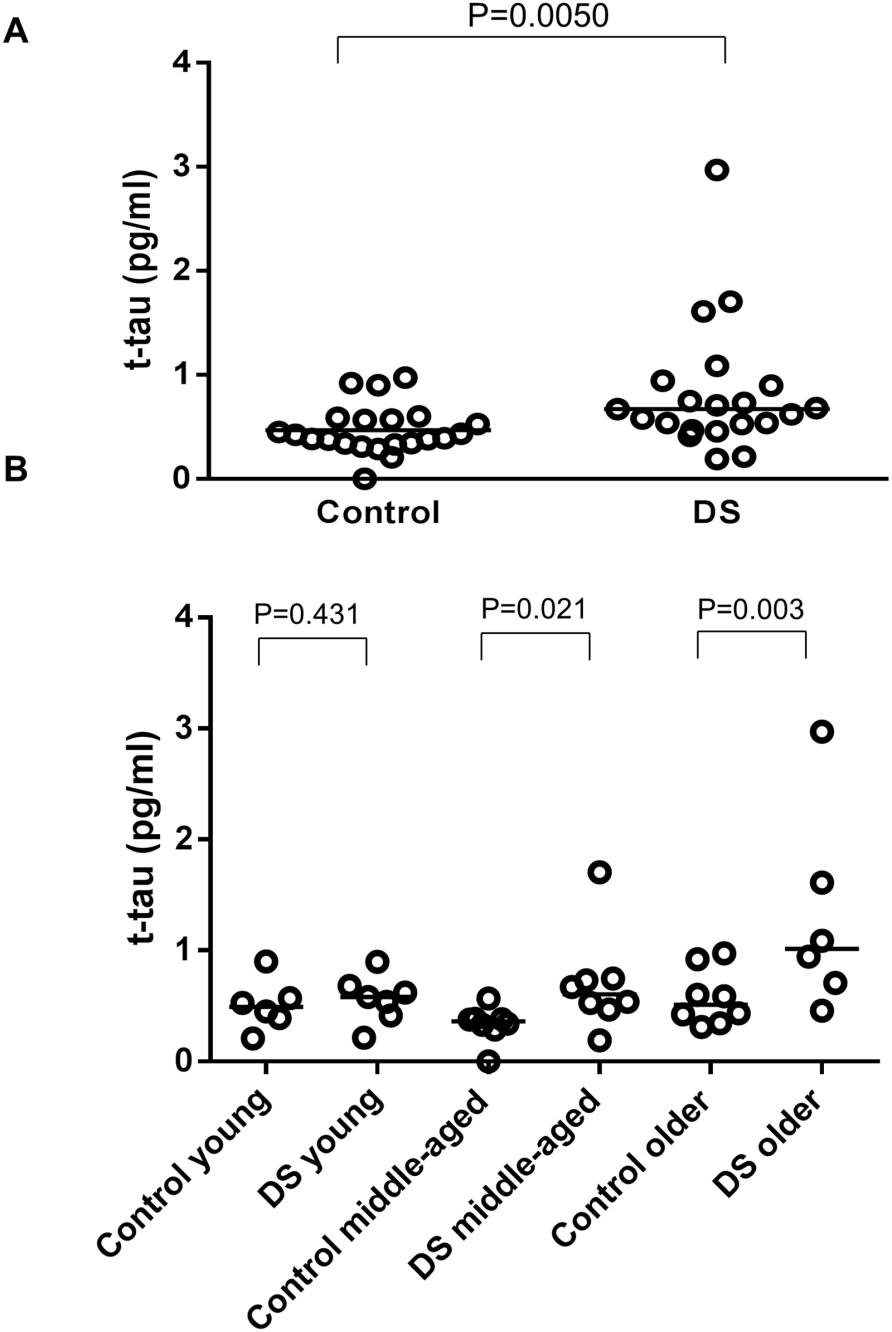
(A) Scatter plot for t-tau level in plasma in the control group (n = 22) and DS group (n = 21). Bars indicate median values. Levels of t-tau in the DS group were significantly higher than those of the control group (P = 0.0050). (B) Scatter plot for t-tau level in plasma in young generation (ages 14–25 years; n = 6 in the control group and 7 in the DS group), middle-aged generation (ages 26–42 years; n = 8 in the control group and 8 in the DS group), and older generation (aged older than 43 years; n = 8 in the control group and 6 in the DS group) generation. Bars indicate median values. Levels of t-tau in the DS group were significantly higher than those of the control group in the middle-aged generation and the older generation (P = 0.021 and 0.003, respectively).

**Fig 2:**
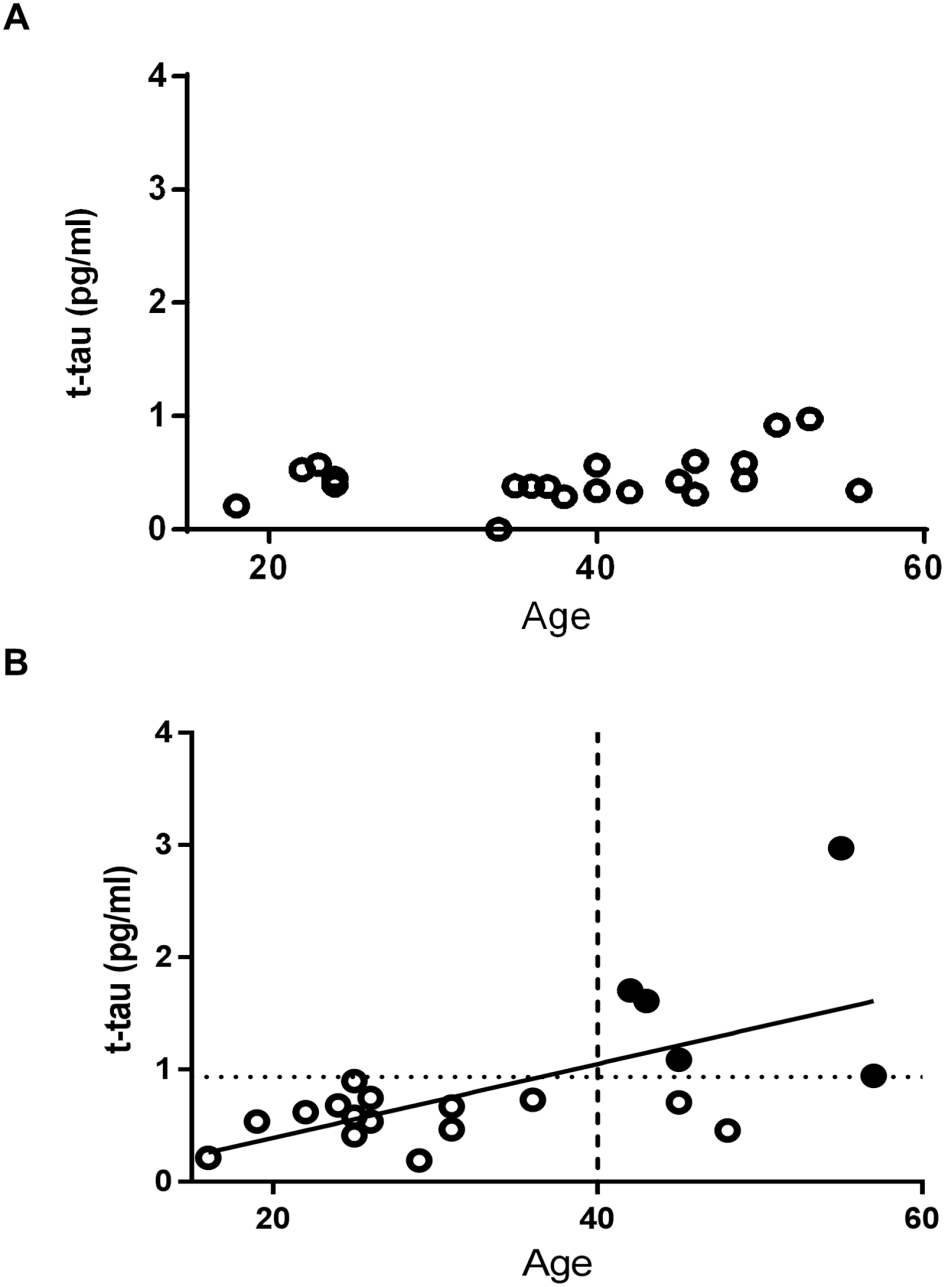
(A) Correlation between t-tau levels and age in the control group. There was no significant relationship. The P-value obtained from Spearman’s rank correlation coefficient test was 0.420. (B) Correlation between t-tau levels and ages in the DS group. A significant positive relationship was found (P = 0.022). Filled circles (black) indicate the DS cases with t-tau levels exceeding the cut-off value (0.934 pg/ml indicated by a dotted horizontal line), estimated from the control group. A dashed vertical line has been drawn to indicate the position of 40 years of age.

The demographic characteristics, cognitive assessments, and concentration of plasma t-tau of participants are summarized in Tables 1 and 2. We found no significant difference in median ages (P = 0.313) and gender frequencies (P = 0.245) between the DS group and the control group. As shown in Fig 1, levels of plasma t-tau were significantly higher in the DS group, compared to the control group (P = 0.0050). When participants were categorized by age into young generation, middle-aged generation, and older generation, the significant elevation of plasma t-tau in the DS group was seen in the middle-aged and older generation, but not in the young generation. There was no significant relationship between ages and levels of plasma t-tau in the control group, while a significant positive relationship between levels of plasma t-tau and ages was observed in the DS group (P = 0.022) (Fig 2A). We set the cut-off value at 0.934 pg/ml based on the mean value and the standard deviation (SD) in the control group (i.e. two SD above the mean value). The DS group had five patients with abnormally elevated plasma t-tau according to this cut-off value. All of them were over the age of 40. (Table 2, Fig 2B)

**Table 1:** Concentrations of plasma t-tau in the control group.

Case	Sex	Age (years)	Comorbid disease or condition	Plasma t-tau (pg/ml)
1	M	14	Epilepsy	0.903
2	M	18	Epilepsy	0.209
3	F	22	Normal	0.530
4	M	23	Anxiety neurosis	0.573
5	F	24	Anxiety neurosis	0.452
6	F	24	Normal	0.391
7	F	34	Normal	0.000
8	M	35	Normal	0.384
9	M	36	Normal	0.383
10	M	37	Normal	0.380
11	F	38	Normal	0.290
12	M	40	Cavernous angioma	0.567
13	F	40	Normal	0.343
14	M	42	Normal	0.331
15	M	45	Diabetes Mellitus	0.424
16	M	46	Normal	0.310
17	F	46	Normal	0.600
18	F	49	Normal	0.436
19	M	49	Normal	0.587
20	F	51	Normal	0.923
21	F	53	Epilepsy	0.974
22	M	56	Normal	0.345
	M:F 12:10	Mean±SD 37.4±12.0		Mean±SD 0.470±0.232

**Table 2:** Cognitive assessments and concentrations of plasma t-tau in patients with DS.

Case	Sex	Age (years)	DSQIID (total scores*)	Social Age (years**)	Plasma t-tau (pg/ml)
1	M	16	10	4.00	0.214
2	F	19	15	4.5	0.538
3	F	22	0	7.42	0.623
4	F	24	4	10.00	0.681
5	F	25	0	7.00	0.899
6	M	25	14	N/A	0.414
7	M	25	2	7.08	0.581
8	F	26	1	4.17	0.537
9	M	26	12	4.92	0.749
10	M	26	0	6.50	0.530
11	M	29	36	4.67	0.193
12	F	31	15	N/A	0.671
13	M	31	2	2.33	0.467
14	M	36	2	8.75	0.733
15	F	42	17	4.17	1.704
16	M	43	10	6.08	1.610
17	F	45	13	1.92	1.088
18	F	45	22	8.33	0.709
19	M	48	33	4.42	0.459
20	F	55	10	2.42	2.973
21	M	57	22	4.08	0.944
	M:F 11:10	Mean±SD 33.1±11.9	Median 10	Mean±SD 5.41±2.26	Mean±SD 0.643±0.493

N/A: not available

*: DSQIID total score indicate the sum total of the scores from part 2 and part 3 [27]

**: Social ages were estimated using social maturity scale revised (S-M). Although data were calculated as units of years and months on this buttery [26], we recalculated those into unit of years for statistical analysis. Social ages in this figure were represented as unit of years.

There were five patients with plasma t-tau levels exceeding the cut-off value (0.934 pg/ml: two SD above the mean of those in the control group) (indicated by gray shading).

**Fig 3:**
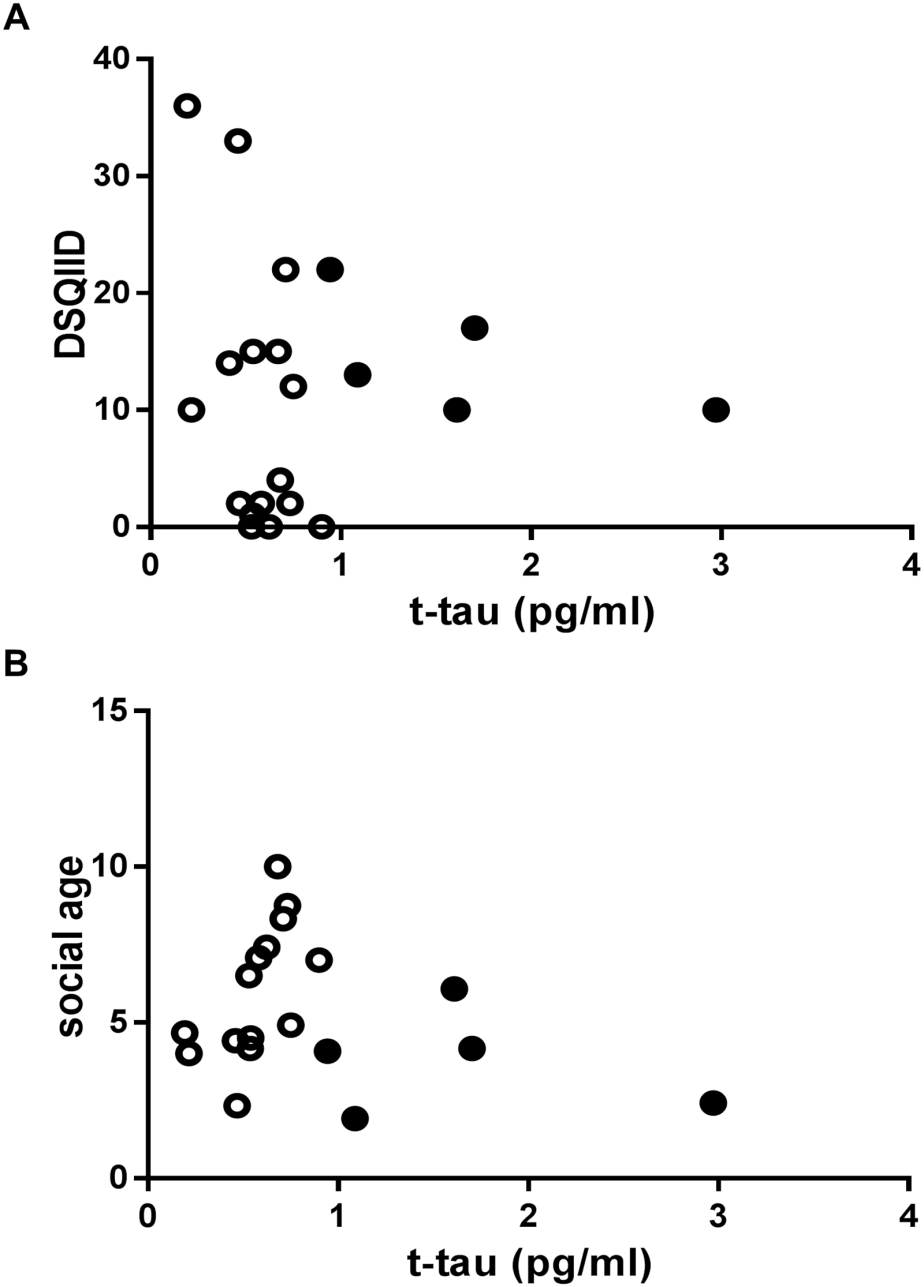
Correlation between t-tau levels and DSQIID score in the DS group(A) and between t-tau levels and social age in the DS group (B). There was no significant relationship. The P value obtained from Spearman’s rank correlation coefficient test was 0.917 in (A) and 0.660 in (B). Filled circles (black) indicate the DS cases with t-tau levels exceeding the cut-off value (0.934 pg/ml indicated by a dotted horizontal line), estimated from the control group.

As shown in Fig 3, plasma t-tau levels in patients with DS were neither significantly related to total DSQIID scores (P = 0.917) nor to social ages (P = 0.898). When we focus on the five patients with abnormally elevated t-tau levels in the DS group, the total DSQIID scores remain within normal range (≤20) in four of the five patients.

## Discussion

The present study showed the increased levels of plasma t-tau in adult patients with DS, whose ages ranged from 16 to 57 years old, compared to those in the age matched control group. This result is consistent with one previous report on plasma t-tau levels in DS using another method called immunomagnetic reduction [22] as well as with one report regarding phosphorylated-tau levels in plasma neuron-derived exosomes in DS [29]. The levels of plasma t-tau did not correlate with age in the control group, in accordance with previous reports [24, 30]. In contrast, levels of plasma t-tau in the DS group increased age-dependently. As shown in Fig 1B, levels of t-tau in the DS group were significantly elevated not only in the older generation but also in the middle aged generation (ages 26–42 years) compared to those in the control group. To best our knowledge, our report is the first study that showed age-dependent elevation of plasma t-tau levels in patients with DS. Considering that elevation of plasma t-tau in patients with AD was reported in several studies [21, 24, 25] our results can be interpreted as being a consequence of AD pathology in DS. This idea would be supported by the fact that abnormal elevation of plasma t-tau was only observed in several patients over 40 years old, which is around the age when tau pathology starts in DS [5]. Taken together, plasma t-tau might have potential as an objective biomarker in the diagnosis of dementia in adult DS. We acknowledge our limitation that many of our patients with DS did not receive tau positron emission tomography. In future studies, such information could help to indicate whether the increase in plasma t-tau was in response to amyloid pathology or if it is a marker that changes prior to amyloid pathology.

On the other hand, the relationship between the levels of plasma t-tau and the scores of cognitive assessments was too weak to reach significance. In addition, the total scores of DSQIID in the five patients with abnormally elevated t-tau were within the normal range, except for one patient. This result is consistent with a report showing that plasma t-tau is not different between DS patients with degeneration (i.e., behavioral and psychological symptoms of dementia or confirmed dementia) and DS patients without degeneration [22]. Our current and the previous results suggest that dementia in DS is difficult to predict with by plasma t-tau alone. We acknowledge insufficient cognitive assessment is a limitation of this study. Future studies should use more extensive cognitive assessments with prospective observations in order to form more definitive comparisons with the levels of plasma t-tau and cognitive decline. Development of more specific biomarkers for AD pathology such as plasma phosphorylated-tau will be useful in future studies.

Leaving aside another obvious limitation of the small sample size, we should mention why the difference of plasma t-tau levels between the DS and the control group was robust enough to reach significance in such a small study. According to the data from Alzheimer’s Disease Neuroimaging Initiative (ADNI) study, the levels of plasma t-tau were significantly higher in the AD group than in the controls. However, there was a considerable overlap between the groups. The effect size that is calculated as difference in means between the two groups divided by the standard deviation of the control group, was 0.45 in the data from ADNI, which is considered as a medium effect size. In contrast, our study showed an effect size of 0.75, which is generally regarded as a large effect size. This discrepancy may be caused by early aging in DS [31]. Patients with DS exhibit signs of premature aging, not only in brain but also in some other organs. For example, premature graying of hair, wrinkling of skin, early menopause, and cataracts are commonly observed in the adult DS population. This phenomenon is thought to be due to both genetic and epigenetic inheritance [32–34]. Taking into account the report that plasma t-tau levels tend to increase to a small extent with age in longitudinal observations [35], plasma t-tau levels may reflect not only AD pathology, but also age related neurodegeneration like primary age-related tauopathy. Considering those facts, such early aging in DS may partly contribute to the elevation of plasma t-tau regardless of AD pathology in the DS group. We should pay attention in future studies to the possibility that such a confounder might consequently cause the difference of plasma t-tau between the DS and control groups to be larger than anticipated.

## Conclusions

This study observed a significant difference of plasma t-tau levels between the DS and healthy control groups. Furthermore, age-dependent correlation of plasma t-tau was only found in the DS group, and not in the control group, suggesting that elevated plasma t-tau reflects AD pathology or neurodegeneration, and therefore, has potential as an objective biomarker to detect dementia in adult DS. Considering the pathological similarity between AD and DS, our results also provide supportive evidence for the idea that plasma t-tau could be a blood-based diagnostic biomarker for AD. In the future, large-scale case-control studies including adequate numbers of DS patients and prospective follow-up are needed to confirm our observations and to elucidate whether elevated plasma t-tau can predict dementia in DS.
